# Ethyl (*Z*)-4-ferrocenyl-2-(4-hy­droxy­anilino)-4-oxobutenoate

**DOI:** 10.1107/S1600536811047763

**Published:** 2011-11-16

**Authors:** Bei-Bei Zhu, Yao-Cheng Shi, Feng-Min Zhang, Li-Min Yuan, Qian-Kun Li

**Affiliations:** a Department of Chemical Engineering, Nantong Vocational College, Nantong 226007, People’s Republic of China; b College of Chemistry and Chemical Engineering, Yangzhou University, 180 SiWangTing Road, Yangzhou 225002, People’s Republic of China; c Testing Center, Yangzhou University, Yangzhou 225009, People’s Republic of China; d Hubei Research Institue of Geophysics Survey and Design, Wuhan 430056, People’s Republic of China

## Abstract

In the title compound, [Fe(C_5_H_5_)(C_17_H_16_NO_4_)], the O=C—C=C—N mean plane is twisted with respect to the mean planes of the benzene and substituted cyclo­penta­dienyl rings by 44.2 (2) and 13.8 (3)°, respectively. Furthermore, the O=C—C=C—N mean plane and the O=C—O(ester) plane make a dihedral angle of 55.5 (6)°. Consistent with this large dihedral angle, the linking C—C bond [1.507 (6) Å] does not show any (delocalized) double-bond character.

## Related literature

For background to the use of enamino­nes and enamine esters in coordination chemistry, supra­molecular chemistry and organometallic chemistry, see: Prokop *et al.* (2001 ▶); Elassar & El-Khair (2003 ▶); Kascheres (2003 ▶); Shi *et al.* (2004 ▶, 2006 ▶, 2008 ▶). For related structures, see: Prokop *et al.* (2001 ▶).
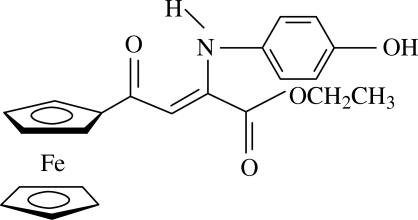


## Experimental

### 

#### Crystal data

[Fe(C_5_H_5_)(C_17_H_16_NO_4_)]
*M* *_r_* = 419.25
Monoclinic, 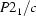

*a* = 15.398 (2) Å
*b* = 11.5131 (15) Å
*c* = 10.9413 (11) Å
β = 95.43 (2)°
*V* = 1931.0 (4) Å^3^
*Z* = 4
Mo *K*α radiation
μ = 0.81 mm^−1^
*T* = 295 K
0.24 × 0.21 × 0.12 mm

#### Data collection

Enraf–Nonius CAD-4 diffractometer
Absorption correction: ψ scan (North *et al.*, 1968 ▶) *T*_min_ = 0.821, *T*_max_ = 0.902
3787 measured reflections
3787 independent reflections
2461 reflections with *I* > 2σ(*I*)
3 standard reflections every 200 reflections  intensity decay: none

#### Refinement

*R*[*F* ^2^ > 2σ(*F* ^2^)] = 0.061
*wR*(*F* ^2^) = 0.172
*S* = 1.08
3787 reflections
237 parameters
H-atom parameters constrained
Δρ_max_ = 0.58 e Å^−3^
Δρ_min_ = −0.86 e Å^−3^

### 

Data collection: *CAD-4 Software* (Enraf–Nonius, 1989 ▶); cell refinement: *CAD-4 Software*; data reduction: *XCAD4* (Harms & Wocadlo, 1995 ▶); program(s) used to solve structure: *SIR2004* (Burla *et al.*, 2005 ▶); program(s) used to refine structure: *SHELXTL* (Sheldrick, 2008 ▶); molecular graphics: *PLATON* (Spek, 2009 ▶) and *WinGX* (Farrugia, 1999 ▶); software used to prepare material for publication: *publCIF* (Westrip, 2010 ▶).

## Supplementary Material

Crystal structure: contains datablock(s) I, global. DOI: 10.1107/S1600536811047763/fj2460sup1.cif

Structure factors: contains datablock(s) I. DOI: 10.1107/S1600536811047763/fj2460Isup2.hkl

Additional supplementary materials:  crystallographic information; 3D view; checkCIF report

## Figures and Tables

**Fig. 1.**
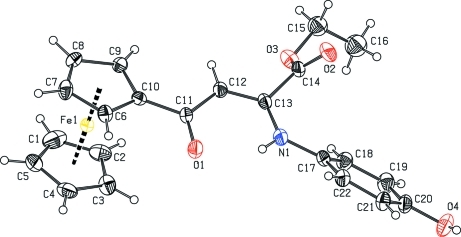
The molecular structure of the title compound, showing the atom-labelling scheme. Displacement ellipsoids are drawn at the 30% probability level.

## supplementary crystallographic information

### Comment

Recently enaminones and related compounds have been used as ligands in coordination chemistry and have been extensively used as versatile synthetic intermediates that combine the ambident nucleophilicity of enamines with the ambident electrophilicity of enones for the preparation of a variety of heterocyclic systems including some natural products and analogues (Elassar & El-Khair, 2003; Kascheres, 2003).

It has been shown that primay amines, Ar^'^NH_2_, react smoothly with β-diketones, ArCOCH_2_COR, to give enaminones, ArCOCH═ C(NHAr^'^)*R*, in good yields (Shi *et al.*, 2004). As part of an ongoing investigation of the chemistry of ferrocenyl enaminones and related compounds (Shi *et al.*, 2006), the title compound, (C_5_H_5_)FeC_5_H_4_COCH═C(NHC_6_H_4_-4-OH)CO_2_CH_2_CH_3_, has been synthesized *via* the reaction of 4-aminophenol with (C_5_H_5_)FeC_5_H_4_COCH_2_COCO_2_CH_2_CH_3_ and structurally characterized.

As noted in the compounds previously reported, the O═ C–C═C–N moiety is planar and the bond lengths indicate electron delocalization (Shi *et al.*, 2004). The O═C–C═C–N plane is twisted with respect to the benzene and substituted cyclopentadienyl rings by 44.2 (2) and 13.8 (3)° whereas the values in an analogous compound are 38.2 (2) and 2.5 (2)° (Prokop *et al.*, 2001). Furthermore, the O═C–C═C–N plane and the O═ C–O plane make a dihedral angle of 55.5 (6)° which is greater than that (48.1 (4)°) of the analogous compound. Consistent with the large dihedral angle between the O═C–C═C–N plane and ester group, the C13–C14 bond, is typical of a single bond (C_sp2_–C_sp2_), and therefore indicates that the ester group is not involved in the conjugation of the O═C–C═C–N moiety. Similarly, the C10–C11 bond suggests that the substituted cyclopentadienyl ring is not involved in the conjugation of the O═ C–C═C–N moiety (Shi *et al.*, 2006).

### Experimental

A mixture of ethyl 4-ferrocenyl-2, 4-dioxobutanate (1.3 g, 4 mmol) and 4-aminophenol (0.43 g, 4 mmol) in 20 ml of absolute ethanol was refluxed for 18 h. After removal of the solvent, the residue was purified by chromatography on silica gel using diethyl ether and dichloromethane (*v*/*v*, 1:10) as an eluant to give the title compound as a purple-red solid (m.p. 412.25-413.65 K, yield 62%). Analysis calculated for C_22_H_21_FeNO_4_: C 63.03, H 5.05, N 3.34%; found: C 63.12, H 5.27, N 3.31%. IR (KBr): 3401 (*m*, HO), 3078 (*m*, HN),1706 (*s*, O═C), 1592 and 1562 (*vs*, *s*, O═C and C═C) cm^-1^. UV (λ_max_, (ε× 10^4^), in DMF): 290 (1.90, *B*-band), 420 (1.37, *K*-band), 574 (0.03, *CT*-band) ^1^H NMR (600 MHz, CDCl_3_, p.p.m.): δ 11.52 (s, HN), 6.88-6.90, 6.79-6.81 (d, d, 2H, 2H, C_6_H_4_), 5.94 (s, 1H, HC), 4.83, 4.52 (s, s, 2H, 2H, C_5_H_4_), 4.23 (s, 5H, C_5_H_5_), 4.17-4.20 (q, 2H, OCH_2_), 1.10-1.13 (t, 3H, CH_3_).

### Refinement

All H atoms were placed at geometrically idealized positions and subsequently treated as riding atoms at 295 K, with C–H = 0.93 (aryl and alkenyl), 0.96 (CH_3_), 0.97 (CH_2_), N–H = 0.86 Å, or O–H = 0.82Å and *U*_iso_(H) values of 1.2*U*_eq_(C, N) or 1.5*U*_eq_(C_methyl_, O).

### Crystal data

**Table:**

[Fe(C_5_H_5_)(C_17_H_16_NO_4_)]	*F*(000) = 872
*M**_r_* = 419.25	*D*_x_ = 1.442 Mg m^−^^3^
Monoclinic, *P*2_1_/*c*	Mo *K*α radiation, λ = 0.71073 Å
*a* = 15.398 (2) Å	Cell parameters from 25 reflections
*b* = 11.5131 (15) Å	θ = 9–15°
*c* = 10.9413 (11) Å	µ = 0.81 mm^−^^1^
β = 95.43 (2)°	*T* = 295 K
*V* = 1931.0 (4) Å^3^	Prism, dark-red
*Z* = 4	0.24 × 0.21 × 0.12 mm

### Data collection

**Table:**

Enraf–Nonius CAD-4 diffractometer	2461 reflections with *I* > 2σ(*I*)
Radiation source: fine-focus sealed tube	*R*_int_ = 0.000
graphite	θ_max_ = 26.0°, θ_min_ = 1.3°
ω/2θ scans	*h* = −18→18
Absorption correction: ψ scan (North *et al.*, 1968)	*k* = 0→14
*T*_min_ = 0.821, *T*_max_ = 0.902	*l* = 0→13
3787 measured reflections	3 standard reflections every 200 reflections
3787 independent reflections	intensity decay: none

### Refinement

**Table:**

Refinement on *F*^2^	Primary atom site location: structure-invariant direct methods
Least-squares matrix: full	Secondary atom site location: difference Fourier map
*R*[*F*^2^ > 2σ(*F*^2^)] = 0.061	Hydrogen site location: inferred from neighbouring sites
*wR*(*F*^2^) = 0.172	H-atom parameters constrained
*S* = 1.08	*w* = 1/[σ^2^(*F*_o_^2^) + (0.0754*P*)^2^ + 1.4205*P*] where *P* = (*F*_o_^2^ + 2*F*_c_^2^)/3
3787 reflections	(Δ/σ)_max_ = 0.001
237 parameters	Δρ_max_ = 0.58 e Å^−^^3^
0 restraints	Δρ_min_ = −0.86 e Å^−^^3^

### Special details

**Table:**

Geometry. All e.s.d.'s (except the e.s.d. in the dihedral angle between two l.s. planes) are estimated using the full covariance matrix. The cell e.s.d.'s are taken into account individually in the estimation of e.s.d.'s in distances, angles and torsion angles; correlations between e.s.d.'s in cell parameters are only used when they are defined by crystal symmetry. An approximate (isotropic) treatment of cell e.s.d.'s is used for estimating e.s.d.'s involving l.s. planes.
Refinement. Refinement of *F*^2^ against ALL reflections. The weighted *R*-factor *wR* and goodness of fit *S* are based on *F*^2^, conventional *R*-factors *R* are based on *F*, with *F* set to zero for negative *F*^2^. The threshold expression of *F*^2^ > σ(*F*^2^) is used only for calculating *R*-factors(gt) *etc*. and is not relevant to the choice of reflections for refinement. *R*-factors based on *F*^2^ are statistically about twice as large as those based on *F*, and *R*- factors based on ALL data will be even larger.

### Fractional atomic coordinates and isotropic or equivalent isotropic displacement parameters (Å^2^)

**Table:**

	*x*	*y*	*z*	*U*_iso_*/*U*_eq_	
C1	1.1956 (4)	0.6697 (5)	0.7793 (6)	0.0691 (18)	
H1	1.2219	0.6929	0.8554	0.083*	
C2	1.1223 (4)	0.5950 (4)	0.7610 (5)	0.0525 (14)	
H2	1.0920	0.5600	0.8208	0.063*	
C3	1.1049 (3)	0.5853 (4)	0.6308 (5)	0.047	
H3	1.0600	0.5425	0.5896	0.056*	
C4	1.1659 (4)	0.6499 (5)	0.5765 (5)	0.0557 (14)	
H4	1.1687	0.6568	0.4923	0.067*	
C5	1.2226 (4)	0.7033 (5)	0.6658 (6)	0.0630 (16)	
H5	1.2692	0.7518	0.6527	0.076*	
C6	1.0226 (3)	0.8596 (4)	0.5648 (4)	0.0393 (11)	
H6	1.0145	0.8506	0.4801	0.047*	
C7	1.0875 (3)	0.9262 (4)	0.6291 (5)	0.0447 (12)	
H7	1.1294	0.9700	0.5941	0.054*	
C8	1.0791 (3)	0.9163 (4)	0.7561 (4)	0.0375 (11)	
H8	1.1144	0.9519	0.8189	0.045*	
C9	1.0067 (3)	0.8421 (4)	0.7710 (4)	0.0342 (10)	
H9	0.9863	0.8197	0.8448	0.041*	
C10	0.9715 (3)	0.8086 (4)	0.6503 (4)	0.0331 (10)	
C11	0.8947 (3)	0.7331 (4)	0.6156 (4)	0.0321 (10)	
C12	0.8355 (3)	0.7087 (4)	0.7078 (4)	0.0349 (10)	
H12	0.8489	0.7360	0.7874	0.042*	
C13	0.7605 (3)	0.6466 (4)	0.6807 (4)	0.0345 (10)	
C14	0.6966 (3)	0.6412 (4)	0.7768 (5)	0.0410 (11)	
C15	0.6789 (4)	0.5986 (5)	0.9858 (5)	0.058	
H15A	0.7165	0.6105	1.0611	0.070*	
H15B	0.6375	0.6622	0.9774	0.070*	
C16	0.6323 (4)	0.4913 (6)	0.9946 (6)	0.073	
H16A	0.5875	0.4858	0.9276	0.109*	
H16B	0.6063	0.4891	1.0709	0.109*	
H16C	0.6719	0.4273	0.9911	0.109*	
C17	0.6719 (3)	0.5155 (4)	0.5385 (4)	0.0377 (11)	
C18	0.6590 (3)	0.4246 (4)	0.6184 (5)	0.0456 (12)	
H18	0.6915	0.4206	0.6944	0.055*	
C19	0.5973 (3)	0.3396 (4)	0.5842 (5)	0.0527 (14)	
H19	0.5866	0.2808	0.6391	0.063*	
C20	0.5514 (3)	0.3418 (4)	0.4682 (5)	0.0500 (13)	
C21	0.5663 (3)	0.4315 (4)	0.3901 (5)	0.0458 (12)	
H21	0.5362	0.4337	0.3123	0.055*	
C22	0.6257 (3)	0.5189 (5)	0.4256 (4)	0.0455 (12)	
H22	0.6339	0.5800	0.3723	0.055*	
Fe1	1.09728 (4)	0.75507 (5)	0.68129 (6)	0.0320 (2)	
N1	0.7364 (2)	0.6016 (4)	0.5704 (4)	0.0428 (10)	
H1N	0.7634	0.6281	0.5109	0.051*	
O1	0.8815 (2)	0.6984 (3)	0.5088 (3)	0.0456 (8)	
O2	0.6228 (2)	0.6770 (3)	0.7586 (4)	0.0609 (10)	
O3	0.7315 (2)	0.6003 (3)	0.8827 (3)	0.0542 (9)	
O4	0.4934 (3)	0.2556 (3)	0.4394 (4)	0.0656 (11)	
H4O	0.4609	0.2743	0.3789	0.098*	

### Atomic displacement parameters (Å^2^)

**Table:**

	*U*^11^	*U*^22^	*U*^33^	*U*^12^	*U*^13^	*U*^23^
C1	0.067 (4)	0.059 (4)	0.075 (4)	0.026 (3)	−0.028 (3)	−0.021 (3)
C2	0.067 (4)	0.043 (3)	0.048 (3)	0.019 (3)	0.009 (3)	0.006 (2)
C3	0.047	0.033	0.060	0.000	0.009	0.019
C4	0.060 (3)	0.059 (4)	0.050 (3)	0.008 (3)	0.019 (3)	−0.011 (3)
C5	0.038 (3)	0.058 (3)	0.095 (5)	0.005 (3)	0.016 (3)	−0.021 (3)
C6	0.035 (2)	0.044 (3)	0.039 (3)	−0.003 (2)	0.004 (2)	0.005 (2)
C7	0.038 (3)	0.036 (3)	0.060 (3)	−0.008 (2)	0.004 (2)	0.011 (2)
C8	0.045 (3)	0.028 (2)	0.039 (3)	−0.005 (2)	0.000 (2)	−0.003 (2)
C9	0.039 (2)	0.026 (2)	0.038 (3)	−0.0017 (19)	0.007 (2)	0.0042 (19)
C10	0.032 (2)	0.032 (2)	0.036 (2)	0.0021 (19)	0.0079 (19)	−0.003 (2)
C11	0.025 (2)	0.036 (3)	0.035 (2)	−0.0023 (18)	0.0025 (17)	0.002 (2)
C12	0.030 (2)	0.039 (2)	0.036 (2)	−0.0015 (19)	0.0020 (19)	−0.001 (2)
C13	0.029 (2)	0.032 (2)	0.043 (3)	0.0004 (19)	0.010 (2)	0.005 (2)
C14	0.032 (3)	0.035 (3)	0.058 (3)	−0.006 (2)	0.015 (2)	−0.005 (2)
C15	0.058	0.058	0.058	0.000	0.006	0.000
C16	0.073	0.073	0.073	0.000	0.007	0.000
C17	0.024 (2)	0.041 (3)	0.048 (3)	0.0017 (19)	0.004 (2)	−0.004 (2)
C18	0.039 (3)	0.042 (3)	0.055 (3)	0.000 (2)	−0.003 (2)	0.000 (2)
C19	0.048 (3)	0.039 (3)	0.071 (4)	−0.005 (2)	0.008 (3)	0.010 (3)
C20	0.028 (2)	0.040 (3)	0.081 (4)	−0.003 (2)	0.001 (3)	−0.009 (3)
C21	0.034 (3)	0.052 (3)	0.049 (3)	−0.007 (2)	−0.004 (2)	−0.001 (3)
C22	0.040 (3)	0.048 (3)	0.049 (3)	−0.009 (2)	0.010 (2)	−0.004 (2)
Fe1	0.0290 (3)	0.0303 (3)	0.0369 (4)	−0.0022 (3)	0.0051 (2)	−0.0034 (3)
N1	0.036 (2)	0.052 (3)	0.042 (2)	−0.0086 (19)	0.0096 (18)	−0.0059 (19)
O1	0.0419 (19)	0.062 (2)	0.0332 (18)	−0.0151 (17)	0.0043 (14)	−0.0024 (16)
O2	0.0313 (19)	0.078 (3)	0.074 (3)	0.0073 (19)	0.0108 (18)	−0.011 (2)
O3	0.049 (2)	0.060 (2)	0.057 (2)	0.0072 (18)	0.0193 (18)	0.0076 (19)
O4	0.053 (2)	0.050 (2)	0.091 (3)	−0.017 (2)	−0.010 (2)	−0.003 (2)

### Geometric parameters (Å, °)

**Table:**

C1—C5	1.401 (9)	C11—O1	1.234 (5)
C1—C2	1.418 (8)	C11—C12	1.449 (6)
C1—Fe1	2.025 (6)	C12—C13	1.367 (6)
C1—H1	0.9300	C12—H12	0.9300
C2—C3	1.430 (7)	C13—N1	1.333 (6)
C2—Fe1	2.059 (5)	C13—C14	1.507 (6)
C2—H2	0.9300	C14—O2	1.208 (6)
C3—C4	1.375 (7)	C14—O3	1.318 (6)
C3—Fe1	2.038 (5)	C15—C16	1.437 (8)
C3—H3	0.9300	C15—O3	1.450 (6)
C4—C5	1.391 (8)	C15—H15A	0.9700
C4—Fe1	2.031 (5)	C15—H15B	0.9700
C4—H4	0.9300	C16—H16A	0.9600
C5—Fe1	2.042 (5)	C16—H16B	0.9600
C5—H5	0.9300	C16—H16C	0.9600
C6—C7	1.396 (6)	C17—C22	1.367 (6)
C6—C10	1.407 (6)	C17—C18	1.390 (6)
C6—Fe1	2.029 (5)	C17—N1	1.424 (6)
C6—H6	0.9300	C18—C19	1.390 (7)
C7—C8	1.412 (7)	C18—H18	0.9300
C7—Fe1	2.053 (5)	C19—C20	1.393 (7)
C7—H7	0.9300	C19—H19	0.9300
C8—C9	1.426 (6)	C20—O4	1.352 (6)
C8—Fe1	2.059 (4)	C20—C21	1.373 (7)
C8—H8	0.9300	C21—C22	1.390 (7)
C9—C10	1.432 (6)	C21—H21	0.9300
C9—Fe1	2.043 (4)	C22—H22	0.9300
C9—H9	0.9300	N1—H1N	0.8600
C10—C11	1.487 (6)	O4—H4O	0.8200
C10—Fe1	2.031 (4)		
			
C5—C1—C2	110.0 (5)	O3—C15—H15B	109.1
C5—C1—Fe1	70.5 (3)	H15A—C15—H15B	107.8
C2—C1—Fe1	71.0 (3)	C15—C16—H16A	109.5
C5—C1—H1	125.0	C15—C16—H16B	109.5
C2—C1—H1	125.0	H16A—C16—H16B	109.5
Fe1—C1—H1	125.1	C15—C16—H16C	109.5
C1—C2—C3	105.0 (5)	H16A—C16—H16C	109.5
C1—C2—Fe1	68.4 (3)	H16B—C16—H16C	109.5
C3—C2—Fe1	68.8 (3)	C22—C17—C18	119.8 (4)
C1—C2—H2	127.5	C22—C17—N1	119.6 (4)
C3—C2—H2	127.5	C18—C17—N1	120.5 (4)
Fe1—C2—H2	126.9	C19—C18—C17	119.7 (5)
C4—C3—C2	108.5 (5)	C19—C18—H18	120.2
C4—C3—Fe1	70.0 (3)	C17—C18—H18	120.2
C2—C3—Fe1	70.4 (3)	C18—C19—C20	120.5 (5)
C4—C3—H3	125.7	C18—C19—H19	119.7
C2—C3—H3	125.7	C20—C19—H19	119.7
Fe1—C3—H3	125.4	O4—C20—C21	123.5 (5)
C3—C4—C5	110.2 (5)	O4—C20—C19	117.8 (5)
C3—C4—Fe1	70.5 (3)	C21—C20—C19	118.7 (5)
C5—C4—Fe1	70.4 (3)	C20—C21—C22	121.0 (5)
C3—C4—H4	124.9	C20—C21—H21	119.5
C5—C4—H4	124.9	C22—C21—H21	119.5
Fe1—C4—H4	125.7	C17—C22—C21	120.3 (5)
C4—C5—C1	106.3 (5)	C17—C22—H22	119.9
C4—C5—Fe1	69.6 (3)	C21—C22—H22	119.9
C1—C5—Fe1	69.2 (3)	C1—Fe1—C6	166.2 (3)
C4—C5—H5	126.8	C1—Fe1—C10	152.8 (3)
C1—C5—H5	126.8	C6—Fe1—C10	40.54 (17)
Fe1—C5—H5	125.9	C1—Fe1—C4	66.9 (2)
C7—C6—C10	108.3 (4)	C6—Fe1—C4	107.1 (2)
C7—C6—Fe1	70.9 (3)	C10—Fe1—C4	128.7 (2)
C10—C6—Fe1	69.8 (3)	C1—Fe1—C3	67.6 (2)
C7—C6—H6	125.9	C6—Fe1—C3	116.3 (2)
C10—C6—H6	125.9	C10—Fe1—C3	108.93 (19)
Fe1—C6—H6	125.0	C4—Fe1—C3	39.5 (2)
C6—C7—C8	108.9 (4)	C1—Fe1—C5	40.3 (2)
C6—C7—Fe1	69.1 (3)	C6—Fe1—C5	127.2 (2)
C8—C7—Fe1	70.1 (3)	C10—Fe1—C5	165.7 (2)
C6—C7—H7	125.6	C4—Fe1—C5	39.9 (2)
C8—C7—H7	125.6	C3—Fe1—C5	67.6 (2)
Fe1—C7—H7	126.8	C1—Fe1—C9	119.6 (2)
C7—C8—C9	107.8 (4)	C6—Fe1—C9	68.80 (18)
C7—C8—Fe1	69.7 (3)	C10—Fe1—C9	41.16 (17)
C9—C8—Fe1	69.0 (2)	C4—Fe1—C9	168.2 (2)
C7—C8—H8	126.1	C3—Fe1—C9	131.33 (18)
C9—C8—H8	126.1	C5—Fe1—C9	151.3 (2)
Fe1—C8—H8	126.7	C1—Fe1—C7	130.1 (2)
C8—C9—C10	106.7 (4)	C6—Fe1—C7	39.98 (18)
C8—C9—Fe1	70.3 (3)	C10—Fe1—C7	67.57 (18)
C10—C9—Fe1	69.0 (2)	C4—Fe1—C7	116.4 (2)
C8—C9—H9	126.6	C3—Fe1—C7	148.2 (2)
C10—C9—H9	126.6	C5—Fe1—C7	107.6 (2)
Fe1—C9—H9	125.7	C9—Fe1—C7	68.12 (18)
C6—C10—C9	108.3 (4)	C1—Fe1—C8	110.5 (2)
C6—C10—C11	123.8 (4)	C6—Fe1—C8	67.93 (19)
C9—C10—C11	128.0 (4)	C10—Fe1—C8	68.23 (18)
C6—C10—Fe1	69.7 (3)	C4—Fe1—C8	149.3 (2)
C9—C10—Fe1	69.9 (3)	C3—Fe1—C8	170.53 (18)
C11—C10—Fe1	126.0 (3)	C5—Fe1—C8	117.5 (2)
O1—C11—C12	122.6 (4)	C9—Fe1—C8	40.70 (17)
O1—C11—C10	119.1 (4)	C7—Fe1—C8	40.17 (19)
C12—C11—C10	118.2 (4)	C1—Fe1—C2	40.6 (2)
C13—C12—C11	121.6 (4)	C6—Fe1—C2	150.3 (2)
C13—C12—H12	119.2	C10—Fe1—C2	118.6 (2)
C11—C12—H12	119.2	C4—Fe1—C2	67.6 (2)
N1—C13—C12	123.8 (4)	C3—Fe1—C2	40.8 (2)
N1—C13—C14	118.1 (4)	C5—Fe1—C2	68.5 (2)
C12—C13—C14	117.7 (4)	C9—Fe1—C2	110.12 (19)
O2—C14—O3	124.5 (4)	C7—Fe1—C2	169.2 (2)
O2—C14—C13	122.5 (5)	C8—Fe1—C2	131.7 (2)
O3—C14—C13	112.9 (4)	C13—N1—C17	128.3 (4)
C16—C15—O3	112.6 (5)	C13—N1—H1N	115.8
C16—C15—H15A	109.1	C17—N1—H1N	115.8
O3—C15—H15A	109.1	C14—O3—C15	118.5 (4)
C16—C15—H15B	109.1	C20—O4—H4O	109.5
